# A Randomized, Double-Blind, Placebo-Controlled Trial: Efficacy of *Opuntia ficus*-*indica* Prebiotic Supplementation in Subjects with Gut Dysbiosis

**DOI:** 10.3390/nu16050586

**Published:** 2024-02-21

**Authors:** Marta Mellai, Marta Allesina, Benedetto Edoardo, Federica Cascella, Vincenzo Nobile, Amelia Spina, Fabio Amone, Vincenzo Zaccaria, Violetta Insolia, Anna Perri, Danilo Lofaro, Francesco Puoci

**Affiliations:** 1 Department of Health Sciences, University of Piemonte Orientale, 28100 Novara, Italy; marta.allesina@uniupo.it (M.A.); federica.cascella@uniupo.it (F.C.); 2 Genomics & Transcriptomics Unit, Center for Translational Research on Autoimmune and Allergic Disease, 28100 Novara, Italy; 3 GIGA-CP Italian Association for Primary Care Gastroenterology, 87036 Rende, Italy; 4 R&D Department, Complife Italia S.r.l., 27028 San Martino Siccomario, Italy; 5 Nutratech S.r.l., Spin-Off of University of Calabria, 87036 Rende, Italy; amelia.spina@nutratechtesting.com (A.S.); fabio.amone@nutratechtesting.com (F.A.); 6 R&D Department, Bionap S.r.l., 95032 Belpasso, Italy; v.zaccaria@bionap.com; 7 Alma Mater Europea, 2000 Maribor, Slovenia; 8 Department of Experimental and Clinical Medicine, Magna Grecia University of Catanzaro, 88100 Catanzaro, Italy; anna.perri@unicz.it; 9 Department of Mechanical, Energy, Management Engineering, University of Calabria, 87036 Rende, Italy; danilo.lofaro@unical.it; 10 Department of Pharmacy, Health and Nutritional Sciences, University of Calabria, 87100 Cosenza, Italy; francesco.puoci@unical.it

**Keywords:** *Opuntia ficus-indica*, prebiotics, fibers, gut, dysbiosis, 16S rDNA, sequencing

## Abstract

Gut dysbiosis refers to an imbalance in gut microbiota composition and function. *Opuntia ficus-indica* extract has been shown to modulate gut microbiota by improving SCFA production in vivo and gastrointestinal discomfort (GD) in humans. The aim of this study was to demonstrate the efficacy of Odilia^TM^ on gastrointestinal health by changing the microbial diversity of species involved in inflammation, immunity, oxidation, and the brain–gut–muscle axis. A randomized, double-blind clinical trial was conducted in 80 adults with gut dysbiosis. The intervention consisted of a 300 mg daily intake of Odilia^TM^ (n = 40) or maltodextrin as a placebo (n = 40), administered for 8 weeks. Intervention effect was evaluated using 16S metagenomics and GIQLI/GSAS scores at baseline, at 4 and 8 weeks. Eight weeks of Odilia^TM^ supplementation positively modulates gut microbiota composition with a significant reduction in the *Firmicutes* to *Bacteroidetes* ratio (*p* = 0.0012). Relative abundances of beneficial bacteria (*Bacteroides* and *Clostridium_XIVa*) were significantly increased (*p* < 0.001), in contrast to a significant reduction in pro-inflammatory bacteria (*p* < 0.001). Accordingly, GIQLI and GSAS scores revealed successful improvement in GD. Odilia^TM^ may represent an effective and well-tolerated treatment in subjects with gut dysbiosis.

## 1. Introduction

Gut microbiota refers to the microbial consortium, primarily bacteria, and then viruses, fungi, and bacteriophages that inhabit the gastrointestinal (GI) tract of humans. In healthy individuals, the gut microbiota is composed of a diverse and balanced community of bacteria that play a crucial role in various aspects of human physiology [1]. An imbalance or disruption in the composition and function of this delicate ecosystem determines gut dysbiosis. This latter is associated with an overgrowth of pathobionts or a decrease in beneficial bacterial species.

Gut dysbiosis can be caused by host-specific factors such as genetics, health status (infections, chronic inflammation), lifestyle habits, environmental factors such as diet (high simple sugars, low fiber content), and xenobiotics (antibiotics, drugs, and food additives) [2].

From a clinical point of view, gut dysbiosis can manifest with a variety of symptoms that may range from mild to more severe. The most common presentations include digestive issues (bloating, gas, and abdominal pain), changes in bowel habits, GI distress (indigestion, pyrosis, and nausea), fatigue, skin issues, mood changes (emotional, psychological symptoms), and weight changes [3].

Gut dysbiosis has been implicated in the development or exacerbation of different pathological conditions, from local (irritable bowel syndrome, inflammatory bowel diseases, and GI infections) to systemic (autoimmune diseases, metabolic, and neurological disorders). Dysbiosis usually triggers an inflammatory response in the gut that may contribute to the chronic inflammation commonly found in several diseases [4].

Lifestyle modifications, dietary changes, and targeted interventions may help restore a healthy balance in the gut microbiota. Particularly, strategies based on dietary changes comprise an increase in fiber consumption and a reduction in processed foods, as well as the use of probiotics (live beneficial bacteria) and prebiotics (nourishment for beneficial bacteria) [5].

Prebiotics are non-digestible compounds naturally found in foods or available as dietary supplements. They contribute to restoring the balance of the gut microbiota by supporting: (i) growth and activity of probiotic bacteria; (ii) digestive health; (iii) immune system; and (iv) metabolism [6,7].

In the last few years, studies have focused on the use of botanicals as sources of prebiotic fibers. Botanical substances deriving from plants could be used for various therapeutic or dietary purposes. They can include either plant parts and extracts, or compounds isolated from them. Among botanicals, increasing attention has been paid to *Opuntia ficus-indica* for its potential prebiotic properties [8].

*Opuntia ficus-indica* (L.) Mill is a perennial succulent tropical or subtropical plant that belongs to the Cactaceae family. Originally from Mexico, it thrives today in the Mediterranean region. The Italian production is mainly concentrated in Sicily, which accounts for 80–90% of the national yield, the Southwest Italian region with a perfect climatic condition for *Opuntia* growth [9].

The fruits (pulp and peel) of the plant contain anthocyanins [10] that can modulate the gut microbiota composition and improve short-chain fatty acid (SCFA) production in mice [11].

The cladodes (cladophylls or phylloclades) are the stems of the *Opuntia* plant that get flattened and covered with spines and multicellular hairs or trichomes. Like other succulent plants, cladodes contain high amounts of water but are also rich in protein, dietary fibers, carbohydrates, antioxidants, minerals, vitamins, and polyphenols [12,13]. The phytocomplex is known in the literature to have different health benefits ranging from topical to systemic activity and has a unique fingerprint due to the presence of prebiotic soluble fibers, flavonoids, and phenolic acids, such as hydroxycinnamic acids (piscidic and eucomic acids), restricted to these plants [14,15].

The cladodes have been used for centuries as food resources and in folk remedies for the skin and internal mucosal epithelial protection, for blood sugar and lipid metabolism, as antioxidant and anti-inflammatory agents, and for other conditions.

There is little clinical trial data on the prebiotic activity of cladodes as a food supplement ingredient, even if in vitro and in vivo studies suggest their beneficial potential [16,17,18,19]. Interestingly, a standardized extract of *Opuntia ficus-indica* (L.) cladodes has been recently demonstrated to improve GI discomfort [20].

In this regard, the present study aimed at testing the efficacy of a standardized extract from *Opuntia ficus-indica* (L.) Mill (Odilia^TM^) on the overall health of 80 adult subjects with gut dysbiosis by modulating the gut microbiota composition of species involved in inflammation, immunity, oxidation, and, for the first time, according to the last findings, on the brain–gut–muscle axis [21]. A balanced randomized, double-blind, placebo-controlled trial (RCT) was designed to test Odilia™ prebiotic supplementation compared to maltodextrins (MDX) as a placebo. The intervention consisted of a 300 mg daily intake of Odilia^TM^ (n = 40) or MDX (n = 40), administered for 8 weeks.

The effect of Odilia™ on the gut microbiota (primary endpoint) was evaluated using 16S metagenomics, whereas clinical outcomes (secondary endpoint) were assessed through gastrointestinal quality of life (GIQLI) and Gastroesophageal Reflux Disease (GERD) symptom assessment scale (GSAS) questionnaires and anthropometric measurement at the three timepoints.

## 2. Materials and Methods

### 2.1. Trial Design

The study was a single-center, randomized (1:1 balanced randomization), double-blind, placebo-controlled, parallel-group trial, conducted at Nutratech S.r.l. (a Complife company) (Rende, Italy) between March 2022 and March 2023. The study participant centers were: Nutratech S.r.l. (Rende, Cosenza, Italy), Center for Translational Research on Autoimmune and Allergic Disease (CAAD, University of Piemonte Orientale, Novara, Italy), and Complife Italia S.r.l. (San Martino Siccomario, Pavia, Italy). In particular, all the clinical study operations were performed at Nutratech S.r.l., metagenomics analysis was performed at CAAD, while clinical data analysis, statistics, and reporting were performed at Complife Italia S.r.l.

This RCT was approved by the Ethics Committee—University of Calabria (protocol code H.E.HU.MP.NMA00.080.05.00_IT0002109/22 approved on 21 March 2022) and registered on the ISRCTN registry (Registration number: ISRCTN14562892, https://doi.org/10.1186/ISRCTN14562892).

All the study procedures were carried out in accordance with the Ethical Principles for Medical Research involving Human Subjects outlined in the World Medical Association’s (WMA) Helsinki Declaration and its amendments. Participants were fully informed about all experimental procedures and signed a written informed consent form prior to participation.

At baseline (visit 1, T0), eligible subjects were divided into two groups (40 subjects each) and randomly allocated to receive the active (Group A) or placebo (Group B) food supplement. The study duration was 8 weeks. Subjects attended clinic visits at baseline after 4 (visit 2, T1) and 8 weeks (visit 3, T2) of product intake. During each visit, stool samples were collected and stored at −80 °C until microbial DNA isolation for metagenomics analysis. All the study parameters were recorded.

### 2.2. Participants

Eligible subjects were all healthy males and females aged between 25 and 50 years old with gut dysbiosis of unknown etiology. Exclusion criteria were clinical history with relevant presence of any disorder that can potentially interfere with the treatment under study, smoking, inability to give informed consent, BMI ≥ 30 kg/m^2^, pregnant and/or breastfeeding, excessive alcohol consumption (>5 drinks/week), history of drug/alcohol/other substances abuse, known food intolerance or food allergy, involvement in a clinical or food study within the previous month, unstable medical diseases (cardiac arrhythmias or ischemia, uncontrolled hypertension, hypotension, diabetes mellitus, kidney failure), history of paralysis or cerebral vascular accident, active cancers or under chemotherapy, other factors limiting the ability of the participant to cooperate during the study. The study further excluded subjects not using the active/placebo supplement for more than one week. Any intake of drugs or food supplements that could interfere with intestinal activity was prohibited.

### 2.3. Intervention

The active treatment arm received one capsule daily containing 300 mg of a commercially available highly concentrated polysaccharide extract from *Opuntia Ficus Indica* (L.) cladode juice (Odilia^TM^, Bionap S.r.l., Piano Tavola Belpasso, CT, Italy), pregelatinized corn starch (87.75 mg), vegetable magnesium stearate (1.35 mg), talc (0.45 mg), and colloidal silica (0.45 mg). The placebo arm received one capsule daily with an identical appearance, containing MDX (300 mg), pregelatinized corn starch (87.75 mg), vegetable magnesium stearate (1.35 mg), talc (0.45 mg), and colloidal silica (0.45 mg). The subjects were asked to return any unused study product, which was used to assess compliance.

### 2.4. Randomization

Subjects were randomly assigned to the active or the placebo product treatment arm (1:1). A restricted randomization list was computer-generated (PASS 11, v11.0.8, PASS, LLC. Kaysville, UT, USA) using the “Efron’s biased coin” algorithm by an external statistician. The study was double-blind, and neither the subjects nor the personnel involved in the study were aware of the active/placebo distribution list. The allocation sequence was concealed by the statistician in sequentially numbered, opaque, and sealed envelopes, reporting the unblinded treatment allocation (based on the subject entry number in the study). A masked allocation sequence was prepared for the staff delivering the intervention based on the randomization number.

### 2.5. Primary and Secondary Efficacy Endpoints

The primary endpoint was the assessment of the intestinal flora by microbial analysis using 16S rRNA gene sequencing at baseline and after 4 and 8 weeks of product use.

The evaluation of the GI health by GIQLI and GSAS validated questionnaires was the secondary endpoint. The study also measured the following anthropometric parameters: body weight, body mass index (BMI), waistline and hip circumferences.

### 2.6. 16S Metagenomics

16S metagenomics analysis was carried out at the Genomics and Transcriptomics Unit, Center for Translational Research on Autoimmune and Allergic Disease (CAAD), University of Piemonte Orientale (Novara, Italy).

#### 2.6.1. DNA Isolation

Stool samples were thawed at room temperature, and microbial DNA was isolated using the QIAmp^®^ PowerFecal^®^ Pro DNA Kit (Qiagen, Hilden, Germany), according to the manufacturer’s instructions. The yield and quality of bacterial DNA were determined on a NanoDrop^TM^ 2000 spectrophotometer (Thermo Fisher Scientifics Inc., Waltham, MA, USA).

To avoid contamination, microbial DNA extraction was performed under sterile conditions using a lamina flow cabinet, sterile reagents, and materials in agreement with good scientific practices.

#### 2.6.2. 16S rRNA Gene Sequencing

Purified DNA samples were subjected to 16S metagenomics analysis to compare the distribution and relative abundance of microbial consortia in stool samples at baseline and after prebiotic or placebo intake at T1 and T2. The analysis was performed using the AD4SEQ Microbiota Solution B Kit, a next-generation sequencing (NGS) in vitro (CE-IVD) molecular test. Polymerase chain reaction (PCR) amplification of the V3–V4–V6 hypervariable regions of the bacterial 16S rRNA gene was obtained by employing degenerated primers, according to the manufacturer’s instructions. PCR products were purified using Agencourt AMPure XP magnetic beads (Beckman Coulter Inc., Brea, CA, USA), and indexes were added in a subsequent step.

The hypervariable V3–V4–V6 regions of the bacterial 16S rRNA gene were amplified according to the manufacturer’s instructions.

The DNA concentration of the libraries was measured using the Qubit^TM^ 1X dsDNA HS Assay Kit (Invitrogen Co., Carlsbad, CA, USA) on a Qubit 4 fluorometer (Invitrogen), and samples were pooled in equimolar concentrations. The final amplicon libraries were sequenced on a MiSeq Illumina^®^ sequencing platform (Illumina, CA, USA) using a MiSeq Reagent Kit v2 Nano cartridge for 2 × 250 paired-end sequencing.

#### 2.6.3. Raw Sequence Processing

Raw sequencing data were processed using MicrobAT (Microbiota Analysis Tool) v1.2.1 software (SmartSeq S.r.l., Novara, Italy) and the Ribosomal Database Project (RDP) database. MicrobAT is a standalone software based on a client/server system. Through a graphical interface developed in Java, the user can load the FASTQ files, download metadata files, and print the reports of the samples.

MicrobAT applies a cleaning of the reads obtained from the FASTQ file using algorithms that remove short sequences (read length < 200 nt) and low-quality sequences (average Phred quality score < 25) [22]. High-quality sequences are then aligned with the reference RDP database v11.4 [23]. During the taxonomic assignment process, only reads with a minimum sequence length that aligned with a reference ≥ 80% and a similarity threshold ≥ 97% were associated with the species taxonomic level. Finally, the software provides absolute and relative abundance tables and three input files (OTU, taxonomy, and metadata) for the subsequent statistical analyses with MicrobiomeAnalyst [24,25].

Rarefaction curves on raw data were evaluated to assess the species richness of the sample as a function of the sequencing depth.

The taxonomic nomenclature at the phylum level covered by the International Code of Nomenclature for Prokaryotes was reported in round brackets [26].

### 2.7. Clinical Endpoints

#### 2.7.1. Anthropometric Measurements

The body weight was measured using a digital scale (with gram precision). The subject’s height, for BMI calculation, was measured using a stadiometer. The waistline and hip circumferences were measured using a flexible meter (with mm precision).

#### 2.7.2. Gastrointestinal Health Assessment

The GIQLI questionnaire contains 36 questions, each with five response categories in the “Likert scale” style (technique for measuring attitude). The score from each question is from 0 (worst) to 4 (best). The responses to questions are summed to give a numerical score ranging from 0 to 144, with a higher score implying a better QoL. Patients with more severe GI symptoms generally reach an average score corresponding to 45 points, compared to the median score of 126 for healthy controls [27,28]. We analyzed answers and evaluated the scores for the following symptoms: Abdominal pain, sense of fullness, sense of swelling, flatulence, eructation, bowel sounds, bowel movements, reflux, constipation, nausea, and heartburn. GSAS is the most complete evaluation scale for GI symptoms [29]. It is a 15-item tool evaluating various aspects, including stress about GI symptoms before and after treatments. GSAS is valid, stable, and sensitive to changes in symptom intensity over time [30].

### 2.8. Statistical Methods

Sample size was calculated with a two-sided 5% significance level and a power of 80% considering a 20% variation of the primary endpoint due to both inter-individual human variability and error in the measurement techniques. A sample size of 40 subjects per group was necessary, given, and anticipated dropout rate by 20%. The sample size was calculated using PASS 11 statistical software (v11.0.8 for Windows) running on Windows Server 2008 R2 Standard SP1 64-bit edition (Microsoft, Redmond, WA, USA).

Bioinformatical analysis of 16S rRNA gene sequencing data was performed at the Genomics and Transcriptomics Unit (CAAD). Statistical analysis of clinical data was assessed at Complife Italia S.r.l. (San Martino Siccomario, Pavia, Italy).

The results reported in this paper are for the PP population and include all the randomized subjects with complete data for all the endpoints and a compliance to treatment equal to or above 87.5% (i.e., less than one week of product use discontinuation).

#### 2.8.1. Analysis of Microbial Communities Using MicrobiomeAnalyst

Statistical analysis regarding variations within the bacterial communities was performed using MicrobiomeAnalyst v2 software using the phyloseq package (Comprehensive Statistical, Visual, and Meta-Analysis of Microbiome data) (https://microbiomeanalyst.ca/, accessed on 8 September 2023). MicrobiomeAnalyst contains a marker data profiling module dedicated to community profiling and comparative analysis based on a 16S rRNA marker gene dataset [31,32].

The software applies a low-count filter to remove features appearing in only one sample (considered artifacts) and features with counts < 15 across all samples.

Bacterial community profiling, with respect to sample groups and timepoints, was assessed through biodiversity analyses. Specifically, alpha-diversity (intra-sample biodiversity) was measured using three indices: Observed OTUs (number of unique OTUs in the sample), Shannon entropy (qualitative and quantitative measure of the community richness, *H*′ ≥ 0), and Simpson (measurement of uniformity between taxa or evenness, 0 ≤ D ≤ 1). Beta-diversity (inter-sample biodiversity) was calculated by Bray–Curtis distance (dissimilarity matrix), and the results were visualized in two plots through PCoA. In the plot, each point represents the entire microbiota of a single sample. The statistical significance of the differences between sample groups was evaluated using permutational ANOVA (PERMANOVA).

LDA LEfSe was used to identify microbial signatures at different taxonomic levels, characterizing each sample group. This method estimates the statistical significance and biological consistency (effect size) of data. The Kruskal–Wallis sum-rank test identifies taxa that are statistically different between sample groups, while LDA calculates the effect size of each differentially abundant feature. Features with *p* < 0.05 and an LDA score > or <2 were considered taxa able to discriminate between sample groups. Finally, *p* values adjusted using the false discovery rate (FDR) for multiple testing < 0.05 were considered statistically significant.

The statistical significance of the F/B ratio between sample groups was calculated using a nonparametric, two-tailed Mann–Whitney U test (unpaired samples) and a Wilcoxon sum rank test (paired samples), as appropriate. *p* values < 0.05 were considered statistically significant.

The statistical analysis was carried out using GraphPad Prism (v8.0.2 for Windows, GraphPad, La Jolla, CA, USA).

#### 2.8.2. Analysis of Clinical Endpoints

The non-parametric Kruskal–Wallis one-way ANOVA on Ranks followed by a Tukey–Kramer’s post hoc test was used to determine whether there was a statistically significant variation over time in the endpoint (intra-group statistical analysis), while the non-parametric Mann–Whitney U test was used to determine whether there was a statistically significant variation in the endpoint between active and placebo groups (inter-group statistical analysis). All statistical analyses were one-sided at a 5% significance level (*p* < 0.05). The statistical analysis was conducted by NCSS 10 (v10.0.7 for Windows, NCSS, Kaysville, UT, USA) running on Windows Server 2008 R2 Standard SP1 64-bit edition (Microsoft, Redmond, WA, USA). The level of significance was reported as follows: * *p* < 0.05, ** *p* < 0.01, and *** *p* < 0.001.

## 3. Results

### 3.1. Subject Characteristics

**Figure 1:**
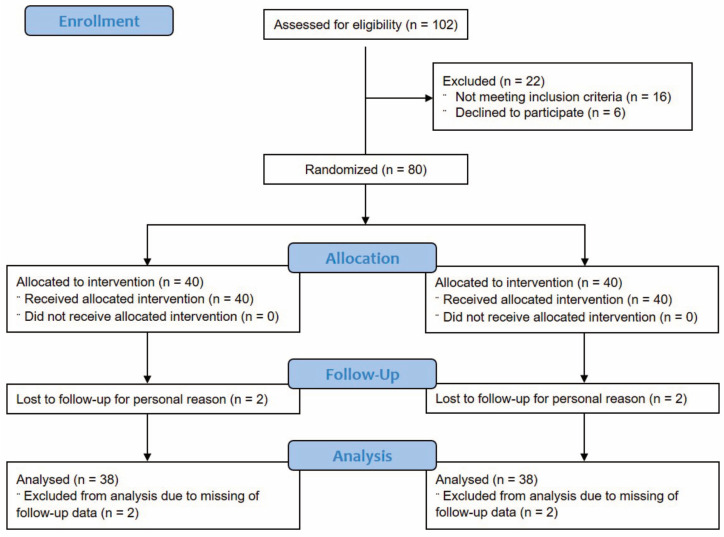
Participants flow diagram.

The study was conducted between January 2022 and March 2023. One hundred and two subjects were screened for eligibility; out of them, 16 did not meet the inclusion criteria, and six declined to participate. The study then successfully randomized eighty (n = 80) subjects suffering from gut dysbiosis; forty (n = 40) subjects were randomized in the active treatment arm (Group A), and forty (n = 40) subjects were randomized in the placebo treatment arm (Group B). The per protocol (PP) population consisted of seventy-six (n = 76) subjects. The number of subjects completing the study was thirty-eight (n = 38) in each treatment arm. The reason for the exclusion from the PP population was related to withdrawing due to personal reasons (n = 2 in the active group and n = 2 in the placebo group). The Odilia^TM^ flowchart diagram is shown in Figure 1.

Both the active and placebo products were well tolerated, and none of the test subjects was lost due to the occurrence of adverse events.

The male-to-female ratio was equal in Group A (42.1% vs. 57.9%) and in Group B (39.5% vs. 60.5%). The mean age (mean ± standard error) was 36.0 ± 1.7 in Group A and 36.9 ± 1.6 in Group B. Additional demographic data at inclusion are reported in Table 1. The demographic and baseline characteristics were not statistically significantly different (*p* > 0.05) between the two sample groups, indicating an unbiased randomization and the absence of covariates.

### 3.2. 16S Metagenomics: Microbial Community Profiling

#### 3.2.1. Data Processing and Quality Control

16S rRNA gene sequencing data quality was assessed by statistic metrics and rarefaction curves. Three sequencing batches were performed, according to the time point, with an average number of reads equal to 107,432 for T0, 117,449 for T1, and 135,683 for T2. All samples had >25,000 good-quality reads, as required by DGPRE 0018191-P-15/06/20181 to consider the results reliable [33].

The analysis of the rarefaction curves (Supplementary Figure S1) as a function of sequencing depth showed that all curves, at each time point, were proximal to saturation, indicating that the richness of the sample has been fully sequenced.

In order to investigate differences in the gut microbiota composition between the active (Group A) and placebo group (Group B), samples were stratified according to the time point and intervention (active or placebo). A total of seven comparisons were assessed: (i) T0 A vs. B; (ii) T1 A vs. B; (iii) T2 A vs. B; (iv) A T0 vs. T2; (v) B T0 vs. T2; (vi) A T0 vs. T1 vs. T2; and (vii) B T0 vs. T1 vs. T2.

#### 3.2.2. Microbial Community Profiling at the Baseline

By MicrobiomeAnalyst, the microbial composition of the two groups at the baseline accounted for six phyla (including one parent taxa), 12 classes (three parent taxa), 14 orders (four parent taxa), 23 families (seven parent taxa), 48 genera (20 parent taxa), and 112 species (42 parent taxa).

**Figure 2:**
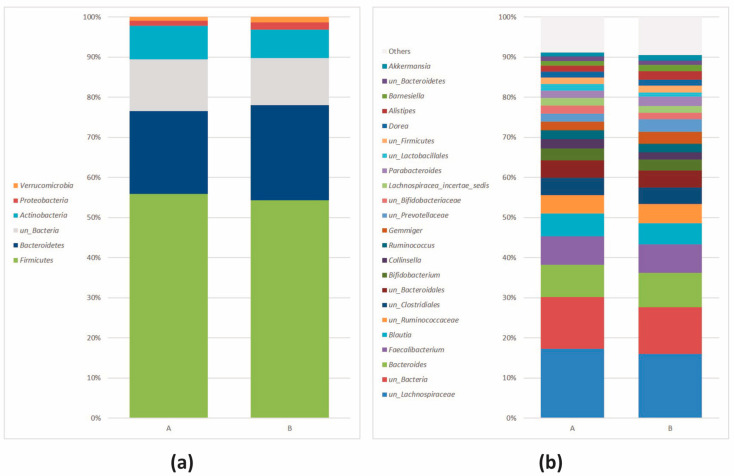
Relative abundance (>1%) at the (**a**) phylum and (**b**) genus level at baseline. Genera < 1% were reported as “Others”. Stacked bar plot.

*Firmicutes* (*Bacillota*) was the most abundant phylum, followed by *Bacteroidetes* (*Bacteroidota*), *Actinobacteria* (*Actinomycetota*), *Proteobacteria* (*Pseudomonadota*), and *Verrucomicrobia* (*Verrucomicrobiota*) (Figure 2a).

The *Firmicutes* to *Bacteroidetes* ratio (F/B ratio), cited in the scientific literature as a hallmark of gut microbiota wellness, was equal to 2.72 and 2.29 (*p* > 0.05) in Group A and Group B, respectively, confirming that both groups display gut dysbiosis.

The abundance profiles of the prevalent genera confirmed the trend observed at the phylum level. Genera belonging to *Firmicutes* (*Bacillota*) (*Fecalibacterium*, *Blautia*, *Ruminococcus*, *Gemmiger*, *Lachnospiracea_incertae_sedis*, *Dorea*) were the most abundant, followed by *Bacteroidetes* (*Bacteroidota*) (*Bacteroides*, *Alistipes*, *Barnesiella*), *Actinobacteria* (*Actinomycetota*) (*Bifidobacterium* and *Collinsella*), *Proteobacteria* (*Pseudomonadota*) (*Gemmiger* and *Alistipes*), and *Verrucomicrobia* (*Verrucomicrobiota*) (*Akkermansia*). The relative abundances, measured as median values, of the most frequent genera (>1%, n = 23) are depicted in Figure 2b. Genera with a relative abundance < 1% were indicated as “Others”.

The biodiversity analysis of the microbial community was evaluated in terms of intra-sample diversity (alpha-diversity) and inter-sample diversity (beta-diversity). Alpha-diversity was assessed by three different indices. Richness was measured using the observed and Shannon indices, whereas evenness was measured with the Simpson index. Beta-diversity was estimated by the Bray–Curtis distance matrix and expressed using the Principal Coordinate Analysis (PCoA). At the baseline, the biodiversity analysis showed a similar intra- and inter-microbial composition at each taxonomic rank (all *p* > 0.05).

Linear discriminant analysis effect size (LDA LEfSe) did not reveal a differential microbial signature, confirming that the two groups were comparable (FDR adjusted *p* value > 0.05).

The two sample groups still remained comparable over time (T1 and T2), as indicated by the biodiversity indices and LDA LEfSe analysis (all *p* > 0.05).

#### 3.2.3. Comparison of the Microbial Diversity in Group A at T0 and T2

**Figure 3:**
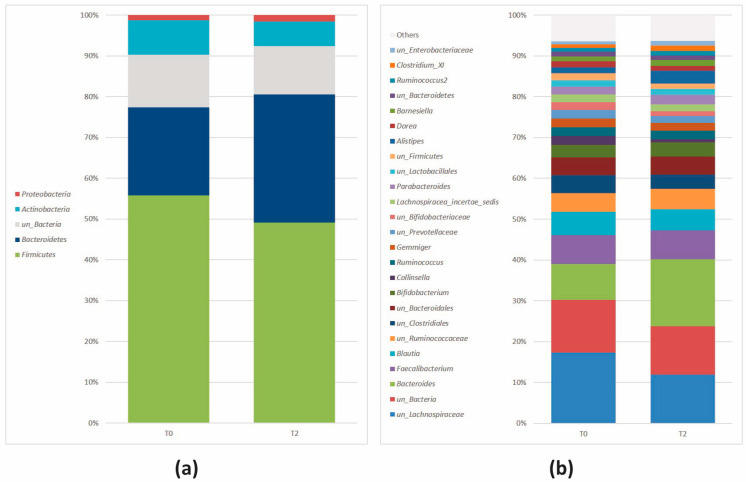
Relative abundance at the (**a**) phylum and (**b**) genus level after 8 weeks. Genera with relative abundance < 1% were reported as “Others”. Stacked bar plot.

MicrobiomeAnalyst software identified 116 taxa across all samples. Five phyla (including one parent taxa) were detected; their relative abundances are described in Figure 3a.

The F/B ratio significantly decreased from 2.72 (T0) to 1.56 (T2) (*p* = 0.0012). *Firmicutes* (*Bacillota*) and *Bacteroidetes* (*Bacteroidota*) were reduced and increased, respectively, leading to a lower value of the F/B ratio, suggestive of a more eubiotic condition.

At the genus level, the software detected 46 taxa across all samples. The relative abundance distribution of the most frequent genera (>1%, n = 25) is reported in Figure 3b. Genera with a relative abundance of <1% were indicated as “Others”. Their abundance profiles were in line with the trend observed at the phylum level, with a reduction in genera belonging to *Firmicutes* (*Bacillota*) and an increase of genera belonging to *Bacteroidetes* (*Bacteroidota*).

**Figure 4:**
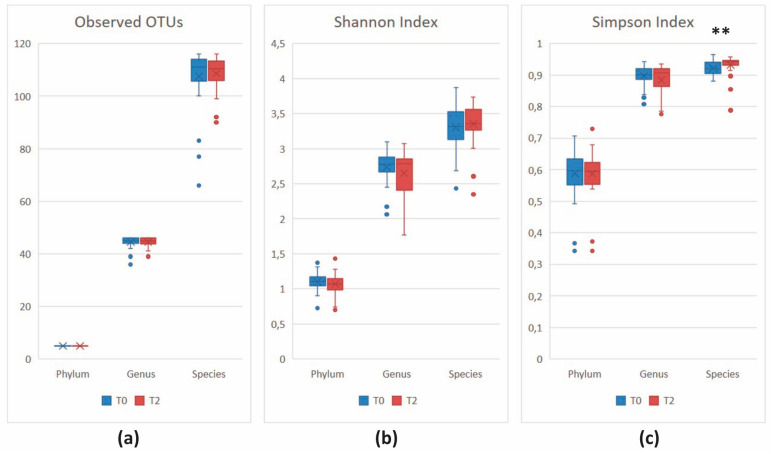
Alpha-diversity analysis at various taxonomic ranks. (**a**) Observed index; (**b**) Shannon index; (**c**) Simpson index. ** *p* < 0.01.

Despite a similar number of unique taxa in the two sample groups (observed index, all *p* > 0.05), at T2, the Shannon index revealed a different distribution of taxa abundances at the genus and species rank (both *p* > 0.05), which is significantly more even at the species level, as shown by the Simpson index (*p* = 0.00553) (Figure 4).

**Figure 5:**
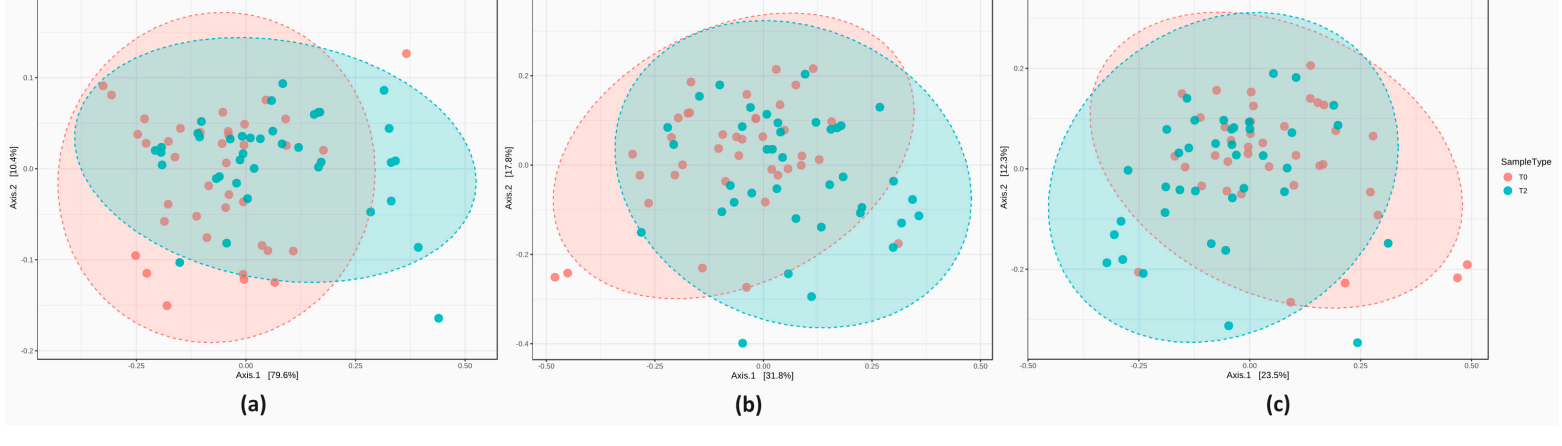
Beta-diversity graphical representation at the (**a**) phylum, (**b**) genus and (**c**) species level (all *p* < 0.001, PERMANOVA).

The PCoA plot revealed a highly significant inter-sample biodiversity at all taxonomic ranks (all *p* < 0.001). After 8 weeks of test supplement, Group A displayed a different microbial structure compared to T0, as indicated by the partial ellipse overlap (Figure 5).

**Figure 6:**
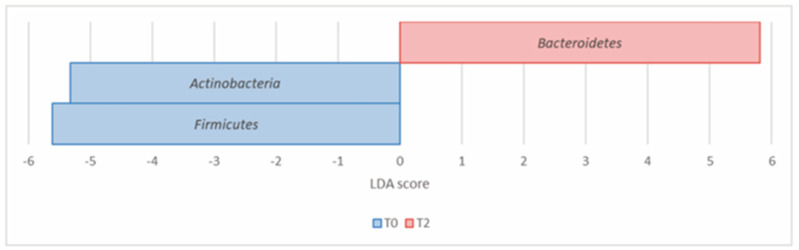
Histogram of the LDA scores computed for differential abundant taxa in Group A at T0 (blue) and T2 (red) at the phylum level.

Remarkably, LDA LEfSe analysis revealed statistically significant differences at all taxonomic ranks (FDR adjusted *p* value < 0.05). At the phylum level, *Bacteroidetes* (*Bacteroidota*) significantly increased from T0 to T2, with a concomitant reduction in *Firmicutes* (*Bacillota*), in agreement with the decreased F/B ratio at T2. *Actinobacteria* (*Actinomycetota*) also reduced from T0 to T2 (Table 2, Figure 6).

**Figure 7:**
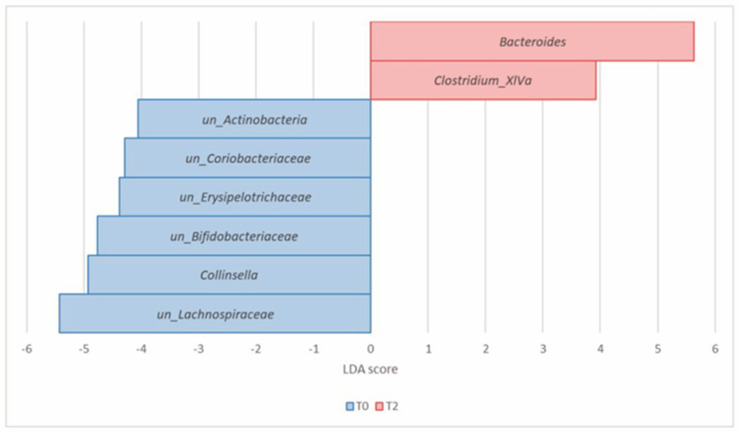
Histogram of the LDA scores computed for differential abundant taxa in Group A at T0 (blue) and T2 (red) at the genus level.

At the genus level, LDA LEfSe identified *Bacteroides* (*Bacteroidetes*) and *Clostridium*_*XIVa* (*Firmicutes*) as significantly enriched at T2, compared to T0. Conversely, *Collinsella*, *unclassified*_*Actinobacteria*, *unclassified*_*Coriobacteriaceae*, *unclassified*_*Bifidobacteriaceae* (all *Actinobacteria*), *unclassified_Lachnospiraceae*, and *unclassified_Erysipelotrichaceae* (*Firmicutes*) were the most significantly decreased genera at T2 (Table 3, Figure 7).

**Figure 8:**
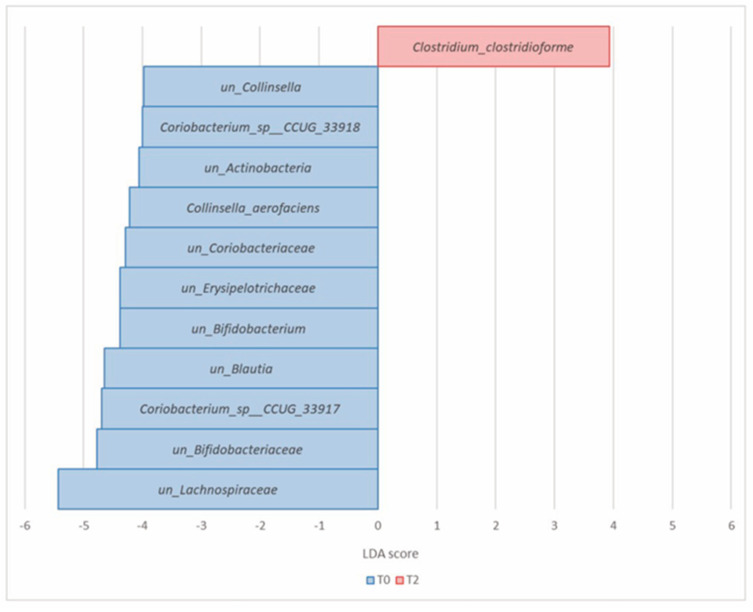
Histogram of the LDA scores computed for differential abundant taxa in Group A at T0 (blue) and T2 (red) at the species level.

At the species level, 12 microorganisms showed significant differential abundances. *Clostridium clostridiforme* (*Firmicutes*) was the unique, significantly enriched species after 8 weeks of prebiotic intake. In contrast, *Collinsella aerofaciens*, *unclassified_Collinsella*, unclassified_*Actinobacteria*, *Coriobacterium_sp__CCUG_33917*, *Coriobacterium_sp__CCUG_33918*, *unclassified_Coriobacteriacae*, *unclassified_Bifidobacteriacae*, *unclassified_Bifidobacterium* (all *Actinobacteria*), *unclassified_Blautia*, *unclassified_Lachnospiraceae,* and *unclassified_Erysipelotrichaceae* (all *Firmicutes*) were the significant prevalent species at T0 (Table 4, Figure 8).

The comparison at the three time points confirmed the data previously shown. The F/B ratio significantly decreased from 2.72 (T0) to 1.77 (T1) (*p* = 0.0296) to 1.56 (T2) (*p* = 0.0012). Moreover, LEfSe revealed an enrichment of *unclassified_Prevotellaceae* (FDR adjusted *p* value < 0.05) going from T0 to T1.

#### 3.2.4. Comparison of the Microbial Diversity of Group B at T0 and T2

**Figure 9:**
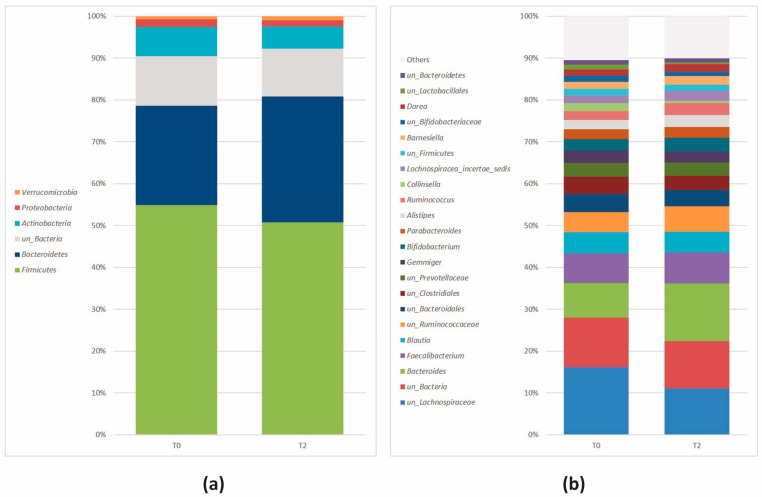
Relative abundance at the (**a**) phylum and (**b**) genus level. Stacked bar plot.

MicrobiomeAnalyst identified a total of 109 taxa. Six phyla (including one parent taxa) were detected across all samples; their relative abundances are described in Figure 9a.

The F/B ratio decreased from 2.29 (T0) to 1.69 (T2), but without statistical significance (*p* > 0.05). Forty-six genera were identified; the relative abundance of the most frequent genera (>1%, n = 22) is reported in Figure 9b. Genera with a relative abundance of <1% are included in “Others”. Their abundance profiles were in line with the trend observed at the phylum level, with a concomitant mild reduction in genera belonging to *Firmicutes* (*Bacillota*) and an increase in genera belonging to *Bacteroidetes* (*Bacteroidota*).

**Figure 10:**
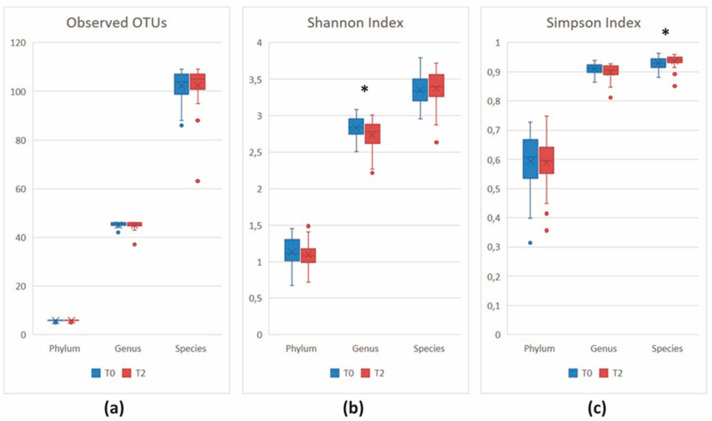
Alpha-diversity analysis at various taxonomic ranks. (**a**) Observed index; (**b**) Shannon index; (**c**) Simpson index. * *p* < 0.05.

The number of unique taxa at the two time points remained similar (observed index, *p* > 0.05). Richness and evenness displayed slight changes at the genus (Shannon index, *p* = 0.03054) and species (Simpson index, *p* = 0.02077) level (Figure 10).

**Figure 11:**
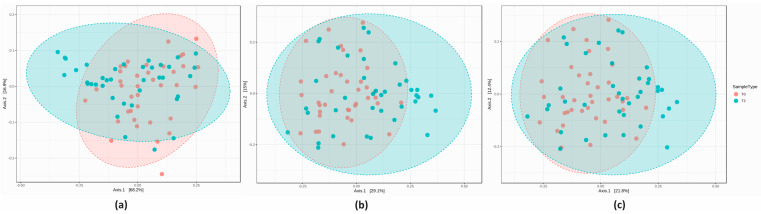
Beta-diversity graphical representation at the (**a**) phylum, (**b**) genus, and (**c**) species level (all *p* < 0.05, PERMANOVA).

The PCoA analysis revealed a significantly different inter-sample biodiversity between T0 and T2 at each taxonomic level (all *p* < 0.05) (Figure 11).

**Figure 12:**
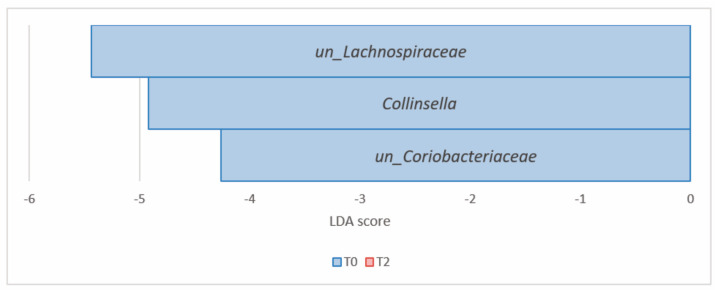
Histogram of the LDA scores computed for differential abundant taxa in Group B at T0 (blue) and T2 (red) at the genus level.

**Figure 13:**
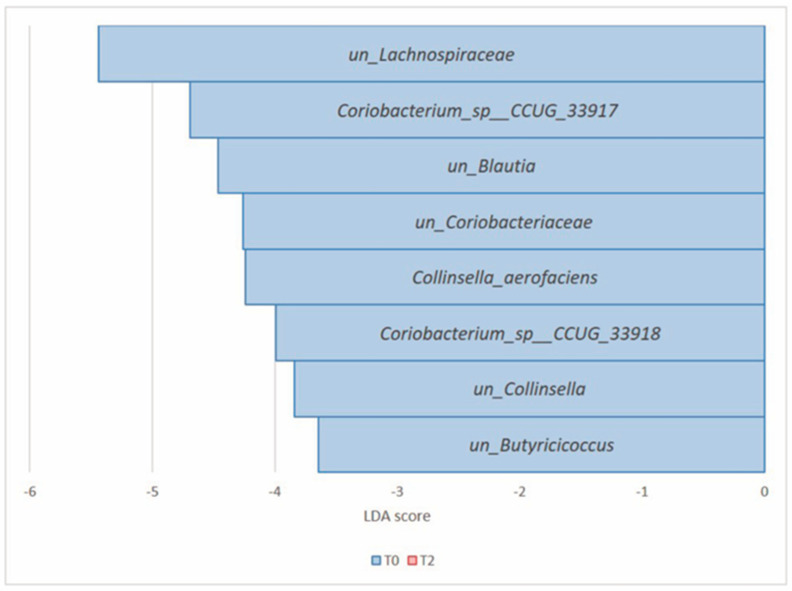
Histogram of the LDA scores computed for differential abundant taxa in Group B at T0 (blue) and T2 (red) at the species level.

LDA LEfSe analysis showed a differential microbial abundance at all taxonomic ranks (*p* value and FDR < 0.05) except for the phylum rank. In particular, the genera *unclassified_Lachnospiraceae* (*Firmicutes*) and *Collinsella*, *unclassified_Coriobacteriaceae* (both *Actinobacteria*) were significantly reduced from T0 to T2 (Table 5, Figure 12). At the species level, the microbial signature at T0 is depicted in Table 6 and Figure 13. Notably, Group B did not show any significant microbial enrichment going from T0 to T2.

The comparison at the three time points revealed a temporary perturbation of the gut microbiota. The F/B ratio varied from 2.29 (T0) to 1.98 (T1) (*p* > 0.05) and 1.69 (T2) (*p* > 0.05). LDA LEfSe analysis did not reveal a differential microbial signature. 

### 3.3. Clinical Endpoints

**Figure 14:**
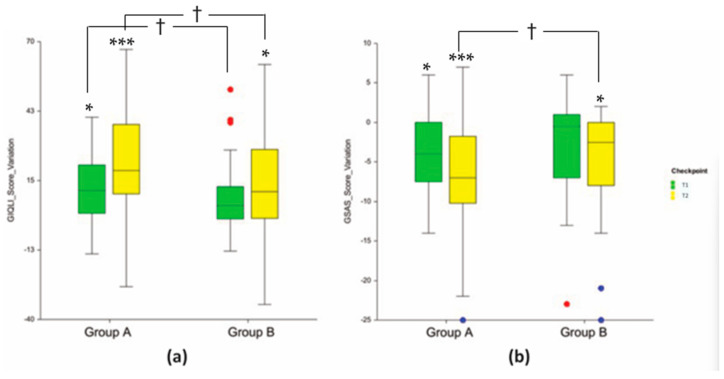
(**a**) GIQLI overall score variation; (**b**) GSAS overall score variation. Above the bars is reported the intragroup (vs. baseline) statistical analysis as follows * *p* < 0.05; *** *p* < 0.001. The intergroup statistical analysis (active vs. placebo) is reported with † upon the bars, as follows † *p* < 0.05.

GIQLI and GSAS questionnaires were used to assess GI health (Figure 14): the higher the GIQLI score, the better the relief of symptoms; the lower the GSAS score, the better the relief of symptoms. The overall median score of GIQLI was statistically significantly improved at T1 (+7.2%, *p* < 0.05) and T2 (+19.6% *p* < 0.001) in Group A, while it was statistically significantly improved only at T2 (+11.8%, *p* < 0.05) in Group B. Similar results were obtained for the GSAS questionnaire. The overall median score of GSAS statistically significantly improved at T1 (−34.6%, *p* < 0.05) and T2 (−53.8% *p* < 0.001) in Group A, while it was statistically significantly improved only at T2 (−40.0%, *p* < 0.05) in Group B. Differences between Group A and B were statistically significant at T1 and T2 (*p* < 0.05) for GIQLI and at T2 (*p* < 0.05) for GSAS. Interestingly, most of the differences between Group A and B were found in the questionnaire’s items related to both physical and emotional/stress components.

Among the physical signs, at T1 and T2, the abdominal pain was statistically significantly improved (+0.7 vs. +0.2 at T1 and +1.1 vs. +0.4 at T2; Group A vs. Group B, respectively) in Group A; heartburn or burning pain inside the chest or breastbone (pyrosis) was statistically significantly improved (−0.8 vs. −0.3; Group A vs. Group B, respectively) at T2 (Table 7).

Among the emotional/stress components towards food intake, troubles with strong burping or belching, the ability to cope with stress, and trouble swallowing food (dysphagia) were improved at T2; while the feeling of fullness in the upper abdomen (abdominal fullness), abdominal noises, fatigue, and early satiety were improved both at T1 and T2. Differences between Group A and B were statistically significant at T2 for troubles with strong burping or belching (+0.7 vs. +0.2, *p* < 0.05; Group A vs. Group B, respectively), the ability to cope with stress (+0.9 vs. +0.5, *p* < 0.05; Group A vs. Group B, respectively), fatigue (+0.9 vs. +0.3, *p* < 0.05; Group A vs. Group B, respectively), and for dysphagia (+0.6 vs. +0.1, *p* < 0.01; Group A vs. Group B, respectively); while differences between Group A and B were statistically significant both at T1 and T2 for abdominal fullness (+0.8 vs. +0.2 at T1 and +1.1 vs. +0.5 at T2, *p* < 0.001 and *p* < 0.05; Group A vs. Group B, respectively), abdominal noises (+0.7 vs. +0.3 at T1 and +0.9 vs. +0.3 at T2, both *p* < 0.05; Group A vs. Group B, respectively), and early satiety (−0.6 vs. −0.2 at T1 and −0.7 vs. −0.2 at T2, *p* < 0.05 and *p* < 0.01, Group A vs. Group B, respectively) (Table 7).

The measured anthropometric parameters (body weight, BMI, waistline, and hip circumference) were not changed. Since a correlation between the gut microbiota and body weight [34,35] was demonstrated, the stability of the anthropometric parameters is a positive finding, suggesting that the body weight and/or other anthropometric parameters did not influence the 16S metagenomics analysis.

## 4. Discussion

The present study aimed to demonstrate that Odilia^TM^ may exert benefits on overall health through a global improvement in the intestinal microbial community and some microorganisms involved in inflammation, immunity, oxidation, and the brain–gut–muscle axis.

Gut dysbiosis can be defined as an imbalance of microbiota subsequent to the loss of the mutualistic relationship within an ecosystem associated with an unhealthy outcome. Hallmarks of gut dysbiosis are a reduction in microbial biodiversity, the loss of beneficial microbial species (i.e., *Bacteroidetes* species and butyrate-producing bacteria such as *Firmicutes*), and the expansion of harmful microbes (pathobionts). These latter are symbionts that, under certain conditions, may become pathogens [19]. An altered F/B ratio is associated with gut dysbiosis, fermentative or putrefactive, in relation to the prevalence, respectively, of *Firmicutes* (*Bacillota*) or *Bacteroidetes* (*Bacteroidota*). In the gut microbial community of a healthy adult, *Bacteroidetes* (*Bacteroidota*) (ranging from 45–50%) and *Firmicutes* (*Bacillota*) (from 42–48%), which both account for about 95%, are expected to be in equilibrium. Values of this ratio between 0.8 and 1.2 can be considered a reference for an eubiotic condition, whereas a ratio greater than 1.2 or below 0.8 is assumed to be a marker of gut dysbiosis. From a translational point of view, the F/B ratio is regarded as a useful biomarker to understand the microbial consortium [36,37].

At baseline, individuals populating Groups A and B share a similar microbial community composition with a taxonomic core signature characterized by a prevalence of pro-inflammatory, immunogenic taxa and potential pathogenic/pathobiont genera. No significant differences among the two sample groups were observed, neither in the active group nor in the placebo group. This finding is in line with expectations, as the subjects enrolled in the study were comparable when related to their internal biodiversity, as a result of a correct randomization process.

Both arms display an increased F/B ratio with a prevalence of *Firmicutes* (*Bacillota*) that is suggestive of a fermentative gut dysbiosis. Accordingly, subjects suffer from clinical symptoms common to this condition, such as constipation and flatulence, as in the literature. Going from T0 to T2, the F/B ratio progressively decreases in both groups. This reduction, which reflects an improvement in the gut microbiota, is greater and statistically significant in Group A than in Group B. We can suppose that this effect may be really referred to as Odilia^TM^ supplementation, as previously reported [38]. In this comparison, the reduction in the F/B ratio did not affect BMI values.

In the Odilia^TM^-treated group, going from baseline to 8 weeks of supplementation, we found a significant improvement in the F/B ratio due to a concomitant reduction in *Firmicutes* (*Bacillota*) and induction of *Bacteroidetes* (*Bacteroidota*) (*p* = 0.0012), relevant changes in the microbial biodiversity, and a differential signature between the two time points. At the end of the intervention, the gut microbiota is significantly depleted by pro-inflammatory and pathobionts (i.e., *Coriobactericeae* spp.) that were prevalent at baseline. At the phylum level, *Actinobacteria* (*Actinomycetota*) and *Firmicutes* (*Bacillota*) were both decreased, as were relevant members at the genus and species levels. Among the former, *Collinsella aerofaciens* is responsible for increased gut permeability by reducing the expression of tight junction proteins and for pro-inflammatory properties that may correspond to an improvement in abdominal discomfort [39,40]. Among *Firmicutes*, the relative abundance of the *Lachnospiraceae* family is decreased. Although members of this family are able to produce beneficial metabolites, an altered abundance of *Lachnospiraceae* is observed in different pathologic conditions, including GI diseases [41,42,43].

At the same time, the active arm is significantly enriched in two beneficial genera, *Bacteroides* (*Bacteroidetes*) and *Clostriudium_XIVa (Firmicutes*), and in the species *Clostridium clostridioforme* (*Firmicutes*), known for anti-inflammatory and immunomodulatory properties. The *Bacteroides* genus is capable of producing the gamma-aminobutyric acid (GABA) neurotransmitter, thus modulating the GABAergic system of the host organism [44,45]. The induction of *Bacteroides* may contribute to the restoration of the gut microbiota composition. The *Clostridium XIVa* genus is a predominant cluster of commensal bacteria in the human gut with beneficial effects on colonic homeostasis. *Clostridium* species have been reported to attenuate inflammation or allergic diseases due to their distinctive biological activities [46]. Metabolites produced from spore-forming bacteria, such as *C. clostridioforme*, are able to stimulate serotonin production from enterochromaffin cells with beneficial effects on the GI and central nervous system via the enteric nervous system [47]. Their cellular components and metabolites (i.e., butyrate, secondary bile acids, and indole propionic acid) also have a probiotic function, mainly by energizing intestinal epithelial cells, strengthening the intestinal barrier, and interacting with the immune system. In particular, the *Clostridium XIVa* cluster exerts its protective functions by producing SCFAs and selectively attracting T reg cells that secrete immunomodulatory cytokines. SCFAs may, in turn, influence gut–brain communication and cognitive function (neuroplasticity), directly or indirectly. Beneficial metabolites, including SCFAs, may also contribute to reducing reactive oxygen species (ROS) and oxidative damage by influencing mitochondrial activities [48,49,50].

In Group B (placebo), going from the baseline to 8 weeks of MDX intake, the F/B ratio is improved but without statistical significance. At 4 weeks of treatment, MDX are responsible for a temporary alteration in the gut microbiota that is totally restored at 8 weeks. LDA LEfSe analysis confirms the pro-inflammatory microbial signature at baseline (as in the active group) and reveals a depletion of *unclassified_Butyricicoccus* (*Firmicutes*), a butyrate producer genus with probiotic potential. MDX does not provide enrichment in any beneficial taxa [51]. Possible MDX effects on the gut microbiota have already been reported in the literature [52]. These alterations refer to changes in the phyla *Firmicutes* and/or *Bacteroidetes* and in the species *Lactobacillus* and/or *Bifidobacterium*, including a bifidogenic effect, which is not found in our group [53].

From a clinical perspective, the results of this study also reveal an improvement in the GIQLI and GSAS questionnaire items related to the emotional/stress components. Among them, abdominal fullness, belching, abdominal noises, the ability to cope with stress, fatigue, dysphagia, pyrosis, and early satiety were improved. These findings are interesting and could be related to the butyrate-producing bacteria. In fact, gut microbiota-derived metabolites, especially SCFAs, have been confirmed to be key molecular mediators in the microbiota–gut–brain axis, and butyrate is one of the bacteria-derived candidates that can link the gut microbiota with brain-derived neurotrophic factor (BDNF) regulation [54,55,56]. Moreover, there is a growing scientific interest in the organ–gut axis and, in particular, the clinical evidence of improvement in coping with stress and fatigue. Stress-associated eating is in line with the so-called brain–gut–muscle axis, in which gut homeostasis seems to be a key factor in human health as the basis for a positive interaction of the intestinal microbiota and the central nervous system, skeletal muscle energy metabolism, and feeding behavior regulation [57].

The daily intake of dietary fiber over the last few centuries has strongly decreased, leading to alternations in the gut microbiota. It is well established that low-fiber diets are linked to a decrease in microbial richness in healthy individuals, with a negative impact on the symbiotic relationship between microbiota and gut, and they may increase the risk of disease onset. On the contrary, high-fiber diets are linked to an improvement in microbiota representatives and health outcomes. Diet integration with prebiotics is nowadays a very common habit when using different food supplements. However, the suggested dosage of fiber intake is commonly about grams of products with a high cutoff threshold (g/day) [58]; in this study, the presence of the whole phytocomplex, intended as the chemical active fingerprint of *Opuntia ficus-indica* L. cladode juice, has proven to achieve clinical outcomes with a very low dose (300 mg/day). Future research should better explore the association between some of the clinical outcomes of the gut–brain axis and gut–muscle axis, together with microbial population changes, as well as a longer period of treatment and a larger population of study. The use of MDX, as expected, was not totally inert but resulted in light microbial population changes, according to the literature data; however, it was used as a placebo since it is the carrier of Odilia^TM^ extract.

In summary, the present RCT demonstrates that subjects with gut dysbiosis may significantly benefit from an 8-week Odilia^TM^ intervention. The improvements are significant and involve both the clinical aspects and the gut microbial biodiversity, which is suggestive of a more favorable consortium for GI and systemic health.

## 5. Conclusions

The present RCT shows that Odilia^TM^ intervention provides significant modifications of the gut microbiota to a more favorable composition that may contribute to improving the overall health of subjects with gut dysbiosis.

These results are promising for individuals struggling with intestinal discomfort who may benefit from Odilia^TM^ consumption.

## Figures and Tables

**Table 1:** Demographic and clinical features at the baseline.

		Group A (n = 38)	Group B (n = 38)	*p* Value ^1^
Age (years)		36.0 ± 1.7	36.9 ± 1.6	0.6918
Sex				
	Male	16 (42.1%)	15 (39.5%)	0.8166
	Female	22 (57.9%)	23 (60.5%)	0.8166
Weight (kg)		73.7 ± 2.1	71.0 ± 2.1	0.3543
Height (cm)		169.8 ± 1.6	168.3 ± 1.6	0.5151
BMI (kg/m^2^)		25.5 ± 0.6	25.0 ± 0.6	0.5135
Hip (cm)		97.3 ± 1.8	95.9 ± 1.7	0.5990
Waist (cm)		83.0 ± 0.1	82.1 ± 1.7	0.7189
GIQLI (score)		91.2 ± 3.4	92.0 ± 3.0	0.8539
GSAS (score)		13.1 ± 1.2	13.2 ± 1.2	0.9628

Continuous variable data are expressed as mean ± standard error; categorical variable data are expressed as count and percentage. ^1^ Two-way, Student’s *t* test for independent samples (Group A vs. Group B). Abbreviations: BMI, body mass index; GIQLI, Gastrointestinal Quality of Life Index; GSAS, GERD Symptom Assessment Scale.

**Table 2:** Results obtained by the LEfSe analysis at the phylum level (FDR adjusted *p* value < 0.05, Kruskal–Wallis test). Negative LDA score: enrichment at T0; positive LDA score: enrichment at T2.

			T0	T2	
Phylum	*p* Value	FDR	Rel Abundance (%)	Rel Abundance (%)	LDA Score
*Actinobacteria (Actinomycetota)*	7.07 × 10^−4^	1.77 × 10^−3^	8.49	5.98	–5.32
*Bacteroidetes (Bacteroidota)*	6.07 × 10^−4^	1.77 × 10^−3^	21.51	31.48	5.81
*Firmicutes (Bacillota)*	1.09 × 10^−2^	1.82 × 10^−2^	55.85	49.10	–5.61

Abbreviations: rel, relative; FDR, false discovery rate; LDA, linear discriminant analysis.

**Table 3:** Results obtained by the LEfSe analysis at the genus level (FDR adjusted *p* value < 0.05, Kruskal–Wallis test). Negative LDA score: enrichment at T0; positive LDA score: enrichment at T2.

				T0	T2	
Phylum	Genus	*p* Value	FDR	Rel Abundance (%)	Rel Abundance (%)	LDA Score
*Actinobacteria* *(Actinomycetota)*	*Collinsella*	8.23 × 10^−5^	1.57 × 10^−3^	2.25	0.66	−4.93
	*un_Actinobacteria*	1.02 × 10^−4^	1.57 × 10^−3^	0.41	0.23	−4.06
	*un_Bifidobacteriaceae*	9.90 × 10^−4^	6.51 × 10^−3^	1.98	1.17	−4.77
	*un_Coriobacteriaceae*	9.90 × 10^−4^	6.51 × 10^−3^	0.74	0.37	−4.29
*Bacteroidetes* *(Bacteroidota)*	*Bacteroides*	3.81 × 10^−4^	4.38 × 10^−3^	8.82	16.38	5.63
*Firmicutes* *(Bacillota)*	*Clostridium_XlVa*	9.34 × 10^−6^	4.30 × 10^−4^	0.04	0.25	3.93
	*un_Lachnospiraceae*	4.82 × 10^−4^	4.43 × 10^−3^	17.27	11.90	−5.43
	*un_Erysipelotrichaceae*	2.59 × 10^−3^	1.49 × 10^−2^	0.67	0.25	−4.38

Abbreviations: un, unclassified; rel, relative; FDR, false discovery rate; LDA, linear discriminant analysis.

**Table 4:** Results obtained by the LEfSe analysis at the species level (FDR adjusted *p* value < 0.05, Kruskal–Wallis test). Negative LDA score: enrichment at T0; positive LDA score: enrichment at T2.

				T0	T2	
Phylum	Species	*p* Value	FDR	Rel Abundance (%)	Rel Abundance (%)	LDA Score
*Actinobacteria* *(Actinomycetota)*	*Coriobacterium_sp__CCUG_33917*	1.60 × 10^−5^	7.92 × 10^−4^	1.24	0.34	−4.69
	*Coriobacterium_sp__CCUG_33918*	2.05 × 10^−5^	7.92 × 10^−4^	0.26	0.07	−4
	*Collinsella_aerofaciens*	4.23 × 10^−5^	1.23 × 10^−3^	0.44	0.13	−4.22
	*un_Actinobacteria*	1.02 × 10^−4^	2.37 × 10^−3^	0.41	0.23	−4.06
	*un_Bifidobacteriaceae*	9.90 × 10^−4^	1.37 × 10^−2^	1.98	1.17	−4.77
	*un_Bifidobacterium*	1.07 × 10^−3^	1.37 × 10^−2^	0.75	0.41	−4.38
	*un_Coriobacteriaceae*	9.90 × 10^−4^	1.37 × 10^−2^	0.74	0.37	−4.29
	*un_Collinsella*	2.35 × 10^−3^	2.73 × 10^−2^	0.31	0.13	−3.98
*Firmicutes* *(Bacillota)*	*Clostridium_clostridioforme*	9.34 × 10^−6^	7.92 × 10^−4^	0.04	0.25	3.93
	*un_Lachnospiraceae*	4.82 × 10^−4^	9.32 × 10^−3^	17.27	11.90	−5.43
	*un_Erysipelotrichaceae*	2.59 × 10^−3^	2.73 × 10^−2^	0.67	0.25	−4.38
	*un_Blautia*	3.07 × 10^−3^	2.97 × 10^−2^	1.82	1.18	−4.64

Abbreviations: un, unclassified; rel, relative; FDR, false discovery rate; LDA, linear discriminant analysis.

**Table 5:** Results obtained by the LEfSe analysis at the genus level (FDR adjusted *p* value < 0.05, Kruskal–Wallis test). Negative LDA score: enrichment at T0; positive LDA score: enrichment at T2.

				T0	T2	
Phylum	Genus	*p* Value	FDR	Rel Abundance (%)	Rel Abundance (%)	LDA Score
*Actinobacteria* *(Actinomycetota)*	*un_Coriobacteriaceae*	6.34 × 10^−5^	1.52 × 10^−3^	0.66	0.30	−4.26
	*Collinsella*	6.63 × 10^−5^	1.52 × 10^−3^	1.87	0.49	−4.92
*Firmicutes* *(Bacillota)*	*un_Lachnospiraceae*	1.56 × 10^−4^	2.39 × 10^−3^	16.22	11.04	−5.44

Abbreviations: un, unclassified; rel, relative; FDR, false discovery rate; LDA, linear discriminant analysis.

**Table 6:** Results obtained by the LEfSe analysis at the species level (FDR adjusted *p* value < 0.05, Kruskal–Wallis test). Negative LDA score: enrichment at T0; Positive LDA score: enrichment at T2.

				T0	T2	
Phylum	Species	*p* Value	FDR	Rel Abundance (%)	Rel Abundance (%)	LDA Score
*Actinobacteria (Actinomycetota)*	*Collinsella_aerofaciens*	1.13 × 10^−5^	9.01 × 10^−4^	0.37	0.08	−4.24
	*Coriobacterium_sp__CCUG_33917*	1.65 × 10^−5^	9.01 × 10^−4^	1.05	0.23	−4.69
	*Coriobacterium_sp__CCUG_33918*	2.74 × 10^−5^	9.97 × 10^−4^	0.20	0.05	−3.99
	*un_Coriobacteriaceae*	6.34 × 10^−5^	1.73 × 10^−3^	0.66	0.30	−4.26
	*un_Collinsella*	2.66 × 10^−3^	3.65 × 10^−2^	0.24	0.13	−3.84
*Firmicutes* *(Bacillota)*	*un_Lachnospiraceae*	1.56 × 10^−4^	3.40 × 10^−3^	16.22	11.04	−5.44
	*un_Butyricicoccus*	2.03 × 10^−3^	3.65 × 10^−2^	0.22	0.15	−3.64
	*un_Blautia*	2.68 × 10^−3^	3.65 × 10^−2^	1.59	1.02	−4.46

Abbreviations: un, unclassified; rel, relative; FDR, false discovery rate; LDA, linear discriminant analysis.

**Table 7:** GIQLI and GSAS questionnaires by items.

	T0		T1		T2	
	Group A	Group B	Group A	Group B	Group A	Group B
**GIQLI**						
Abdominal pain	1.9 ± 0.2	2.4 ± 0.2	2.6 ± 0.2 * (+0.7; +36.8%) *	2.6 ± 0.2 (+0.2; +8.3%)	3.0 ± 0.2 *** (+1.1; +57.9%) *	2.9 ± 0.2 (+0.4; +20.8%)
Abdominal fullness	1.9 ± 0.2	2.3 ± 0.2	2.7 ± 0.2 ** (+0.8; +42.1%) ***	2.5 ± 0.2 (+0.2; +8.7%)	3.0 ± 0.2 *** (+1.1; +57.9%) *	2.8 ± 0.2 (+0.5; +21.7%)
Belching	2.4 ± 0.2	2.7± 0.2	2.7 ± 0.2 (+0.3; +12.5%)	2.8 ± 0.2 (+0.1; +3.7%)	3.1 ± 0.2 * (+0.7; +29.2%) *	2.9 ± 0.2 (+0.2; +7.4%)
Abdominal noises	2.2 ± 0.1	2.4 ± 0.2	2.9 ± 0.2 ** (+0.7; +31.8%) *	2.7 ± 0.2 (+0.3; +12.5%)	3.1 ± 0.2 *** (+0.9; +40.9%) *	2.7 ± 0.2 (+0.3; +12.5%)
Coping with stress	2.3 ± 0.1	2.1 ± 0.2	2.7 ± 0.1 (+0.4; +17.4%)	2.3 ± 0.2 (+0.2; +9.5%)	3.2 ± 0.1 *** (+0.9; +39.1%) *	2.6 ± 0.1 (+0.5; +23.8%)
Fatigue	1.5 ± 0.2	1.9 ± 0.2	2.2 ± 0.1 * (+0.6; +46.7%)	2.3 ± 0.1 (+0.4; +21.0%)	2.4 ± 0.2 *** (+0.9; +60.0%) *	2.2 ± 0.2 (+0.3; +15.8%)
Dysphagia	3.2 ± 0.2	3.5 ± 0.1	3.4 ± 0.2 (+0.3; +6.2%)	3.6 ± 0.1 (+0.1; +2.9%)	3.8 ± 0.1 ** (+0.6; +18.7%) **	3.6 ± 0.1 (+0.1; +2.9%)
**GSAS**						
Pyrosis	1.3 ± 0.2	0.8 ± 0.2	0.9 ± 0.1 (−0.4; −30.8%)	0.7 ± 0.2 (−0.2; −12.5%)	0.5 ± 0.1 *** (−0.8; −61.5%) **	0.5 ± 0.1 (−0.3; −37.5%)
Early satiety	1.0 ± 0.2	0.9 ± 0.2	0.4 ± 0.1 ** (−0.6; −60.0%) *	0.7 ± 0.2 (−0.2; −22.2%)	0.3 ± 0.1 *** (−0.7; −70.0%) **	0.7 ± 0.2 (−0.2; −22.2%)

Continuous variable data are expressed as mean ± standard error; in brackets is reported both the variation and the percentage variation. * *p* < 0.05; ** *p* < 0.01; *** *p* < 0.001. Abbreviations: GIQLI, Gastrointestinal Quality of Life Index; GSAS, GERD Symptom Assessment Scale.

## Data Availability

The data presented in this study are available on request from the corresponding author. The data are not publicly available since they are the property of the sponsor of the study (Bionap S.r.l., 95032 Piano Tavola Belpasso, CT, Italy).

## Supplementary Materials

The following supporting information can be downloaded at: https://www.mdpi.com/article/10.3390/nu16050586/s1, Figure S1: Rarefaction curves describing sequencing depth. All curves reached the plateau, indicating that the sequencing was able to identify all taxa present in the sample.
