# Supplementary figures and images for: A Randomized, Double-Blind, Placebo-Controlled Trial: Efficacy of Opuntia ficus-indica Prebiotic Supplementation in Subjects with Gut Dysbiosis
Source: Nutrients. 2024 Feb 21;16(5):586. doi: 10.3390/nu16050586 (PMC10934938; doi:10.3390/nu16050586)

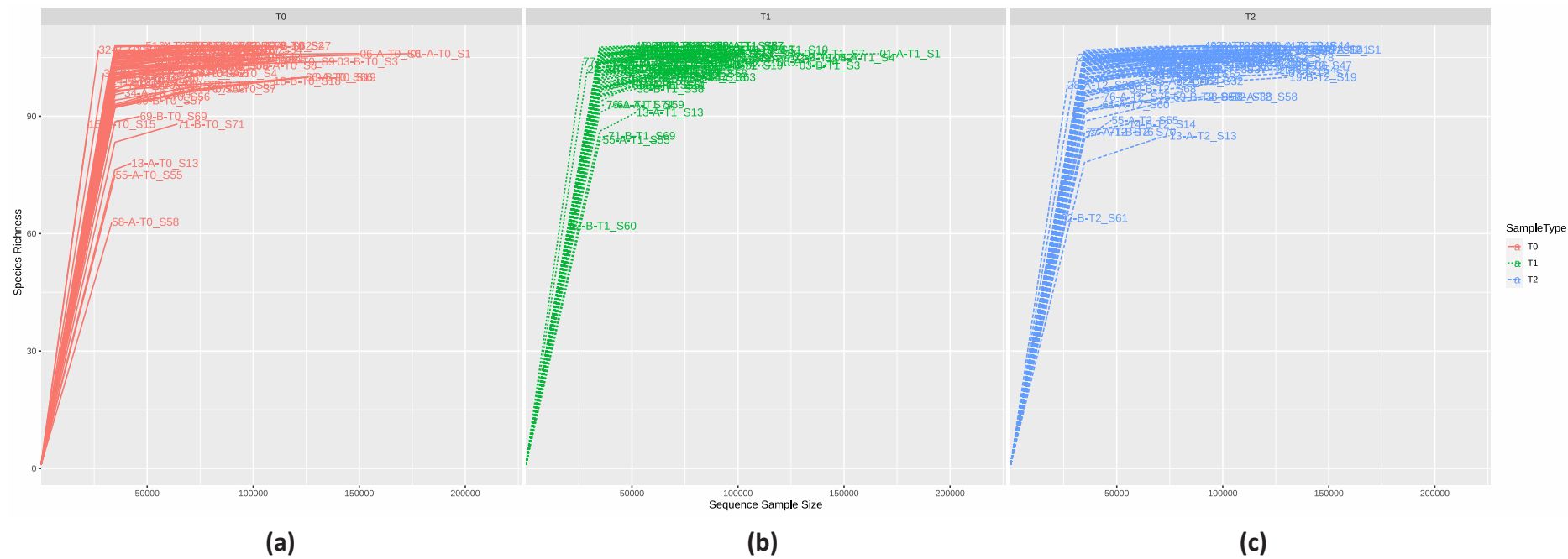

Supplementary material - Figure S1

Supplement: Supplementary file 1 [file nutrients-16-00586-s001.zip › nutrients-2839498-supplementary.pdf]
